# TET3-overexpressing macrophages are a unifying pathogenic feature with therapeutic potential in chronic inflammatory diseases

**DOI:** 10.1172/JCI198802

**Published:** 2025-11-03

**Authors:** Shojiro Haji, Yoshihiro Ogawa

**Affiliations:** Department of Medicine and Bioregulatory Science, Graduate School of Medical Sciences, Kyushu University, Fukuoka, Japan.

## Abstract

Increased activation of the NLRP3 inflammasome in immune cells, including macrophages, has been implicated in the pathogenesis of multiple chronic inflammatory diseases. Targeted depletion of macrophages has been explored as a cross-disease therapeutic strategy, but without subtype-specific markers, this strategy risks elimination of macrophages with homeostatic functions. In this study, Liu et al. identified a subpopulation of pathogenic macrophages, referred to as Toe-Macs, which are characterized by overexpression of the DNA demethylase TET3 in metabolic dysfunction–associated steatohepatitis (MASH), non–small cell lung cancer (NSCLC), and endometriosis. When induced into the disease microenvironment, Toe-Macs produced proinflammatory cytokines and chemokines. Selective elimination of Toe-Macs attenuated disease progression without any discernible side effects in mouse models of MASH and NSCLC. These findings highlight the role of Toe-Macs in the pathogenesis of chronic inflammatory diseases and provide a rationale for exploring TET3 as a therapeutic target.

## NLRP3 inflammasome activation of macrophages in inflammatory diseases

The nucleotide-binding domain, leucine-rich repeat and pyrin domain–containing 3 (NLRP3) inflammasome is a multifunctional intracellular protein complex, the activation of which is primarily observed in immune cells, including macrophages, thus leading to the production of proinflammatory cytokines, such as IL-1β, and induction of pyroptosis through the activation of caspase-1 (1). The above process plays a critical role in the initiation and development of several chronic inflammatory diseases, such as metabolic dysfunction–associated steatohepatitis (MASH) (2), non–small cell lung cancer (NSCLC) (3), and endometriosis (4). NLRP3 and IL-1β inhibitors have been used to treat diseases caused by inflammasome activation, but their immunosuppressive effect has been a major concern because they increase the risk of infection (5). Therefore, there is a need for the development of treatments with fewer immunosuppressive effects.

Recently, pathogenic macrophages, such as those positive for the innate immune receptor TREM2, have been described across several chronic inflammatory diseases (2). Therapeutic targeting of pathogenic macrophages might enable cross-disease strategies applicable to related pathologies in which these cell types are implicated. However, therapeutic targeting of pathogenic macrophages has been challenging, given their functional heterogeneity in the disease microenvironment (DME) (6), as well as the overlapping surface markers expressed among various populations of macrophages and the lack of specific markers to facilitate accurate identification of the macrophage subtype. Moreover, macrophages alter their surface markers and functional properties in response to disease-specific environments (7). Indeed, currently available therapies designed to eliminate pathogenic macrophages, such as a CSF1 receptor inhibitor, predominantly modulate broad myeloid axes rather than directly target these defined subsets, and such interventions have been reported to deplete homeostatic macrophages (8). Accordingly, identification of molecular features specifically associated with pathogenic macrophages and development of strategies for their selective elimination are of paramount importance.

TET3 belongs to the three-member TET family dioxygenases, which also include TET1 and TET2. TET proteins facilitate DNA demethylation by oxidizing 5-methylcytosine to 5-hydroxymethylcytosine. TET proteins can also function by directly recruiting chromatin-modifying complexes independently of catalytic activity (9). For instance, TET2-deficient macrophages display elevated NLRP3 activity with increased secretion of proinflammatory cytokines (10). A recent study by Lv et al. identified pathogenic macrophages characterized by TET3 expression in human endometriosis lesions; transformation of these macrophages was driven by TGF-β1 and MCP1 derived from the DME (11). Pathogenic TET3-expressing macrophages have also been correlated with increased production of cytokines and metastasis in prostate cancer (12).

## Selective elimination of Toe-Macs attenuates disease progression

**Figure 1:**
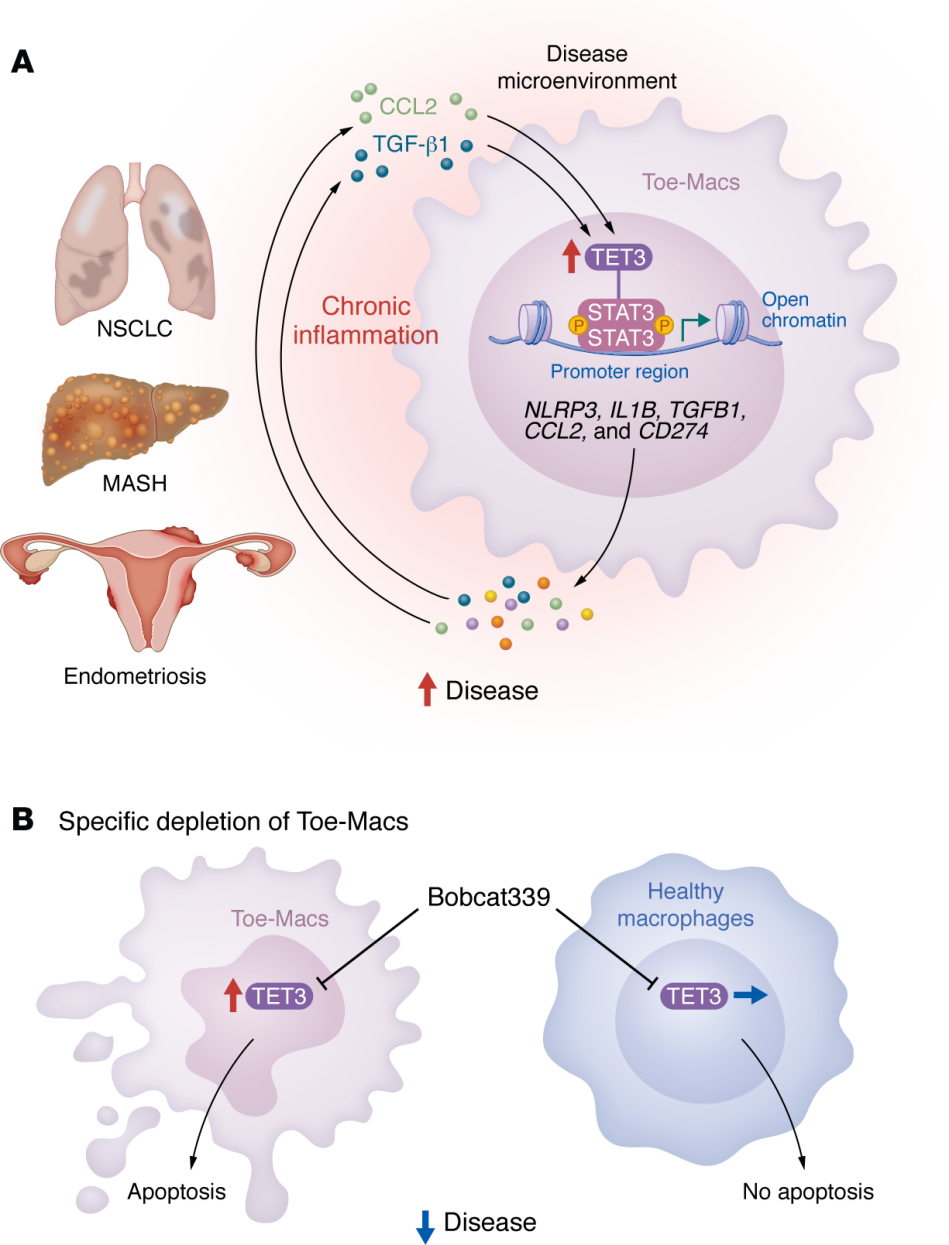
Toe-Macs contribute to the pathogenesis of chronic inflammatory diseases. (**A**) Liu et al. reported that overexpression of TET3 in macrophages is induced by TGF-β1 and CCL2 derived from the DME of MASH, NSCLC, and endometriosis. TET3 is recruited to the promoter regions of *NLRP3*, *IL1B*, *TGFB1*, *CCL2*, and *CD274* via interaction with phosphorylated STAT3 and induces an open chromatin conformation to upregulate the expression of NLRP3, IL-1β, TGF-β1, CCL2, and PD-L1 epigenetically, thereby leading to the exacerbation of these chronic inflammatory diseases. In addition, CCL2 and TGF-β1 released from Toe-Macs could increase TET3 expression through a positive feedback loop. (**B**) Bobcat339, a TET3-specific degrader, does not induce apoptosis in healthy macrophages but selectively trigger apoptosis in Toe-Macs. Therefore, selective elimination of Toe-Macs by using Bobcat339 could mitigate disease progression with reduced side effects.

In this issue of the *JCI*, Liu et al. sought to identify pathogenic macrophages that can be selectively depleted in MASH, NSCLC, and endometriosis, three diseases that share aberrant NLRP3 signaling as a common pathogenic mechanism (13). The authors confirmed that exposing both human peripheral blood monocyte–derived macrophages (MDMs) and mouse bone marrow–derived macrophages (BMDMs) to TGF-β1 or CCL2, two cytokines that are increased in the microenvironment of MASH, increased the expression level of TET3. Conversely, overexpression of TET3 in macrophages induced TGF-β1 and CCL2 production. Taken together, these observations suggest that TET3 upregulates the expression levels of CCL2 and TGF-β1 in macrophages via a positive feedback mechanism (Figure 1A). Additionally, TET3 overexpression sensitized macrophages to NLRP3 activation and IL-1β production. The authors demonstrated that TET3, when recruited to the promoter region via interaction with phosphorylated STAT3, induced an open chromatin conformation, either dependently or independently of its catalytic activity, thereby upregulating the expression of its five target genes *NLRP3*, *IL1B*, *TGFB1*, *CCL2*, and *CD274*. Furthermore, they found that Bobcat339, a synthetic cytosine derivative, selectively degraded TET3 in a von Hippel-Lindau (VHL) E3 ubiquitin ligase–dependent manner, likely via a molecular glue–like mechanism (Figure 1B).

Through a combination of single-cell/single-nucleus RNA-Seq data analysis and IHC studies of liver tissue samples obtained from patients with MASH and two mouse models of MASH, Liu et al. detected pathogenic macrophages characterized by TET3 overexpression (hereafter referred to as Toe-Macs) in MASH livers, which express NLRP3, IL-1β, TGF-β1, CCL2, and IL-6. In two mouse models, selective depletion of Toe-Macs through myeloid-specific *Tet3* knockout or pharmacological treatment with Bobcat339 markedly decreased lipid accumulation and fibrosis (13).

Similarly, in lung tissue samples obtained from patients with NSCLC and a mouse NSCLC model, the authors identified tumor cells expressing high levels of TGF-β1 and CCL2, as well as Toe-Macs expressing NLRP3, IL-1β, TGF-β1, CCL2, IL-6, and PD-L1. Either myeloid-specific *Tet3* ablation or Bobcat339 treatment attenuated lung cancer progression. Interestingly, eliminating Toe-Macs resulted in a significant increase in tumor-infiltrating CD8^+^ T cells expressing higher amounts of granzyme B (GrB), suggesting that TGF-β1 derived from Toe-Macs suppresses GrB expression, in alignment with prior reports (14).

Finally, Liu et al. demonstrated an increase in Toe-Macs in endometriotic lesions compared with eutopic endometrium. As in the mouse models of MASH and NSCLC, Toe-Mac depletion or Bobcat339 treatment in a mouse model of endometriosis reduced disease burden (11). These observations, taken together, suggest that Toe-Macs play a critical role in the establishment and maintenance of an immunosuppressive microenvironment (summarized in Figure 1).

## Conclusions and future studies

The report by Liu et al. (13) underscores the pivotal role of Toe-Macs in the pathogenesis of MASH, NSCLC, and endometriosis, thereby offering a rationale for investigating TET3 as a potential therapeutic target. This discovery extends the seminal work by Lv et al. (11) from the same research group, which demonstrated that Toe-Macs are induced by factors originated from the DME and contribute to the pathogenesis of endometriosis. Furthermore, the findings of Liu et al. indicate that selective elimination of these cells could mitigate disease progression.

This study’s use of human and mouse models for several diseases strengthens the reliability and relevance of the results, thereby offering strong translational potential. Given that Toe-Macs are conserved between mice and humans, the data suggest their importance in the pathogenesis of chronic inflammatory diseases. Moreover, the integration of single-cell RNA-Seq analysis and IHC studies enables the identification of pathogenic macrophages without specific surface molecules. This integrative strategy could facilitate the identification of novel pathogenic macrophage subpopulations that remain undefined by conventional surface markers. Of particular importance is that Bobcat339’s specificity for TET enzymes spares healthy macrophages involved in resolving disease, promoting tissue regeneration and even protecting against infection. Therefore, selective targeting of the pathogenic macrophages could provide a disease-agnostic, rather than disease-specific, treatment strategy with reduced risk of infection and minimal negative effects on tissue repair. In addition, intravenous or oral administration of Bobcat339 showed high bioavailability without discernible side effects in mice. These findings will be critical for clinical translation on a long-term basis.

Although Liu et al. (13) exhibited a range of strengths in their study, certain areas still require further elucidation. Liu et al. (13) showed that TGF-β1 and CCL2 increased TET3 expression at both the mRNA and protein levels. The authors also demonstrated that STAT3 recruited TET3 to its five target genes, thereby regulating their expression via epigenetic mechanisms. Given the critical roles played by the five genes in promoting inflammation and immunosuppression, the ability of TET3 to coordinately regulate their expression via interaction with the shared adaptor protein STAT3 may represent a particularly effective and advantageous mechanism for pathogenic macrophages. However, the detailed molecular mechanisms underlying the TGF-β1– and CCL2-dependent regulation of TET3 expression and STAT3-mediated TET3 recruitment remain unclear. Future work elucidating these mechanisms could provide important insights into the regulatory mechanisms governing TET3 expression and function in pathogenic macrophages during chronic inflammatory diseases. In addition, using the LysM-Cre strain to achieve a myeloid-specific knockout of *Tet3*, as Liu et al. did, might mask specific effects related to the lack of TET3 in macrophages, as LysM is also expressed in other myeloid lineage cells, such as granulocytes and DCs (15, 16).

In conclusion, Liu et al. (13) provided evidence that Toe-Macs are induced by DME-derived TGF-β1 or CCL2 and constitute a common subset of pathogenic macrophages that could be selectively targeted as a single population, owing to their shared susceptibility to TET3 reduction in MASH, NSCLC, and endometriosis. Furthermore, the clinical relevance extends beyond MASH, NSCLC, and endometriosis, since the insights gained from understanding the role of Toe-Macs could provide clues to the treatment approaches for other chronic inflammatory diseases in which Toe-Macs may be involved. Future studies should focus on clarifying the generalizability of these findings and on developing combination therapies that can lead to improved patient outcomes.

## Funding support

Japan Society for the Promotion of Science (JSPS) KAKENHI (JP22H04993, to YO).
Secom Science and Technology Foundation (to YO).
Uehara Memorial Foundation (to YO).
JSPS KAKENHI (JP25K11647, to SH).
Takeda Science Foundation (to SH).

## Version 1 11/03/2025

Electronic publication
